# Correction: Central venous pressure and dynamic indices to assess fluid appropriateness in critically ill patients: A pilot study

**DOI:** 10.1371/journal.pone.0315741

**Published:** 2024-12-10

**Authors:** Chiara Prezioso, Roberta Trotta, Erika Cavallo, Federica Fusina, Elena Malpetti, Filippo Albani, Rosalba Caserta, Antonio Rosano, Giuseppe Natalini

Fig 1 is incorrect. The authors have provided a corrected version here.

**Fig 1:**
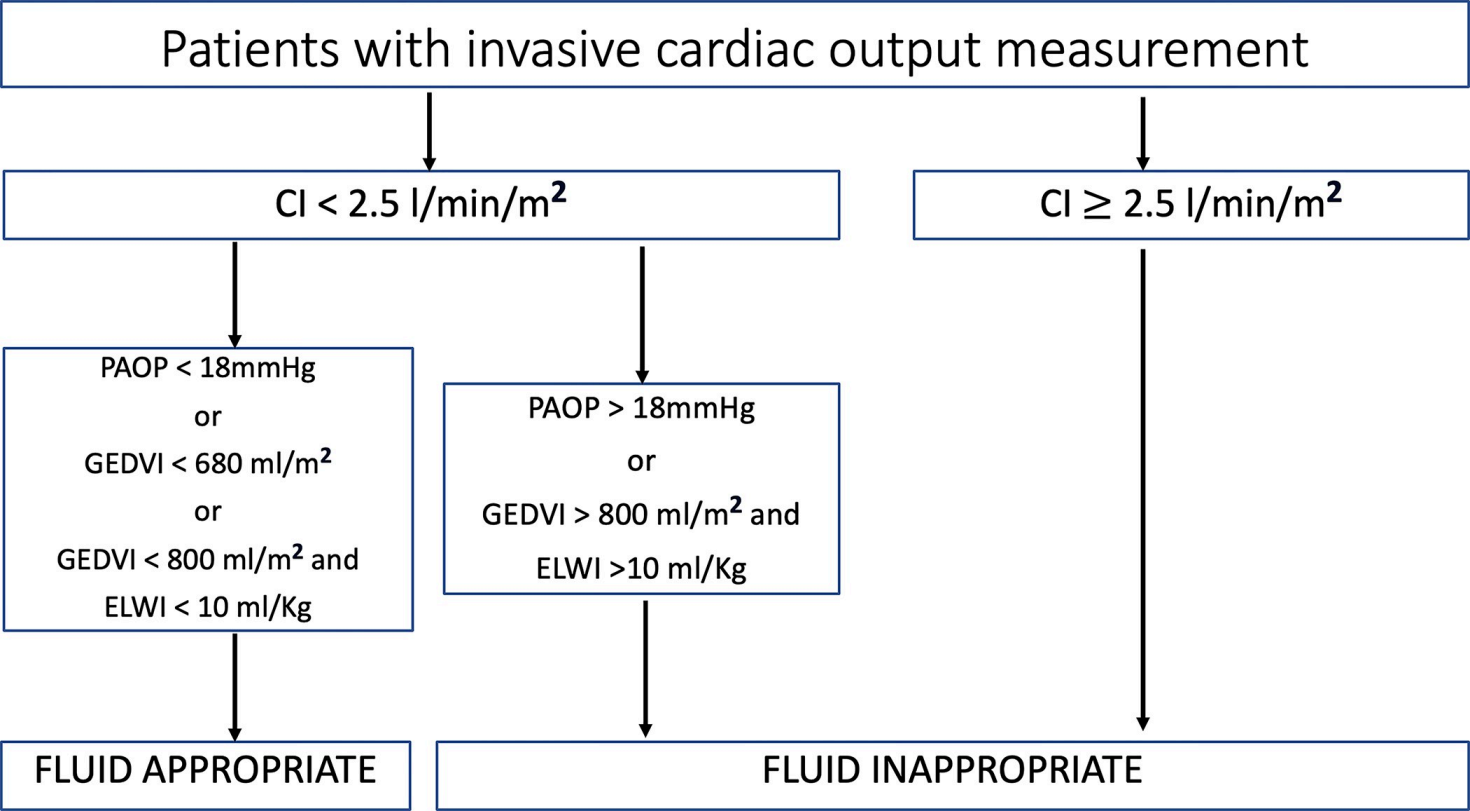
Definition of fluid appropriateness.

Abbreviations: CI = cardiac index, PAOP = pulmonary artery occlusion pressure, GEDVI = global end diastolic volume index, ELWI = extravascular lung water index.
